# Significant Inter-Test Reliability across Approximate Number System Assessments

**DOI:** 10.3389/fpsyg.2016.00310

**Published:** 2016-03-08

**Authors:** Nicholas K. DeWind, Elizabeth M. Brannon

**Affiliations:** Department of Psychology, University of Pennsylvania, PhiladelphiaPA, USA

**Keywords:** Weber fraction, approximate number system, reliability, number sense, numerical cognition, numerical comparison task

## Abstract

The approximate number system (ANS) is the hypothesized cognitive mechanism that allows adults, infants, and animals to enumerate large sets of items approximately. Researchers usually assess the ANS by having subjects compare two sets and indicate which is larger. Accuracy or Weber fraction is taken as an index of the acuity of the system. However, as Clayton et al. (2015) have highlighted, the stimulus parameters used when assessing the ANS vary widely. In particular, the numerical ratio between the pairs, and the way in which non-numerical features are varied often differ radically between studies. Recently, Clayton et al. (2015) found that accuracy measures derived from two commonly used stimulus sets are not significantly correlated. They argue that a lack of inter-test reliability threatens the validity of the ANS construct. Here we apply a recently developed modeling technique to the same data set. The model, by explicitly accounting for the effect of numerical ratio and non-numerical features, produces dependent measures that are less perturbed by stimulus protocol. Contrary to their conclusion we find a significant correlation in Weber fraction across the two stimulus sets. Nevertheless, in agreement with Clayton et al. (2015) we find that different protocols do indeed induce differences in numerical acuity and the degree of influence of non-numerical stimulus features. These findings highlight the need for a systematic investigation of how protocol idiosyncrasies affect ANS assessments.

## Introduction

Adult humans can perceive the number of items in large sets without counting, an ability known as approximate enumeration (Dehaene, 1997). This non-verbal number sense has been termed the approximate number system (ANS). The ANS can be observed in adults from cultures without symbolic counting systems (Pica et al., 2004), in human infants that have not yet acquired language (Xu and Spelke, 2000; Brannon, 2002), in other primates (Cantlon and Brannon, 2007), and in many other taxa (Agrillo et al., 2006; Scarf et al., 2011). As a result, The ANS has been described as a core cognitive system with deep evolutionary and developmental roots (Feigenson et al., 2004).

Unlike symbolic number systems, which allow people to represent quantities precisely and to appreciate number on a linear scale, the ANS supports fuzzy representations of quantity and discrimination is limited by Weber’s Law. Despite these fundamental differences between the ANS and symbolic number systems, a prominent theoretical perspective is that the ANS is foundational for symbolic mathematics (Dehaene, 1997; Wynn, 1998; Gelman and Gallistel, 2004). Convergent findings support this perspective. First acuity of the ANS has been found to correlate with performance in symbolic mathematics, although some studies have failed to replicate that result (for review and meta-analysis see Chen and Li, 2014). In addition to correlational studies a few studies have found that training the ANS yields benefits for symbolic mathematics (Park and Brannon, 2013, 2014; Hyde et al., 2014). Consistent with these findings, severe dyscalculiacs have worse ANS acuity compared to age matched controls (Piazza et al., 2010).

The entire enterprise of studying the relationship between the ANS and mathematics, however, would be severely jeopardized if measures of ANS were found to be unreliable. ANS acuity is typically measured by presenting subjects with a non-symbolic numerosity comparison task in which two arrays of dots are simultaneously and briefly presented on a computer screen and participants are asked to indicate which array contains more dots. However, a wide variety of non-numerical features such as the total area of the items, the area of an individual item, the density of items within the array, and the area of the total stimulus can co-vary with changes in number. This presents a problem for researchers interested in assessing the ability to discriminate number independently of visual features. Currently there is no universally accepted best practice for generating stimuli that control for non-numerical stimulus features (for a review see Dietrich et al., 2015). Instead a wide variety of stimulus and task parameters are used across experiments. If these varying protocols all tap the same underlying cognitive system, then we should see solid inter-test reliability across stimulus sets. However, two groups have presented evidence that performance is uncorrelated across stimulus set (Clayton et al., 2015; Smets et al., 2015). Clayton et al. (2015) present evidence that performance is uncorrelated when individuals are given the same numerical comparison task with two commonly used stimulus sets (Halberda et al., 2008; Gebuis and Reynvoet, 2011). Smets et al. (2015) similarly show that performance is uncorrelated between a stimulus set based on Dehaene et al. (unpublished manuscript) and a subset of the Gebuis and Reynvoet (2011) stimulus set in which multiple visual features, the area subtended by the entire array (convex hull), the total area of the items, individual item size, and density (total area divided by convex hull), are incongruent to number. These studies present a significant problem for measuring ANS acuity and the authors argue that the findings may challenge the construct validity of the ANS itself; indeed, what is a hypothesized cognitive mechanism if we cannot consistently measure it?

We recently pioneered a novel modeling approach to ANS tasks that we argue can shed further light on these important issues (DeWind et al., 2015). Accuracy as used by Clayton et al. (2015), although straightforward, is not necessarily the best dependent measure of ANS acuity, because it is affected not only on the performance of the participant but also on the idiosyncrasies of the stimuli chosen. For example, if in one protocol we present 3:1 numerical ratios participants will perform very well, whereas in another we present 11:10 ratios they will be at chance performance. The mean accuracy on these two tasks might show a very low or zero correlation, but we should not take this as evidence against an underlying perceptual representation of number. Similarly but more subtly, the relationship between number and non-numerical features may introduce noise or suppress variance in accuracy. If a participant is sensitive to the density of items while making numerical judgments, but in one protocol numerical ratio is congruent with density (i.e., the denser arrays are always the more numerous) the participant will perform well, where as in a protocol where number and density are always incongruent they will perform poorly, thus suppressing the correlation in accuracy between protocols.

These examples are extreme to make the point, but it is true that both the numerical and non-numerical ratios differ in the two protocols Clayton et al. (2015) tested, and so the extra variance in accuracy caused by these differences will necessarily negatively influence inter-test reliability. Our modeling approach explicitly accounts for the effects of numerical ratio and non-numerical ratio. Indeed, we previously found that the model provides relatively stable coefficients even when non-numerical features are all congruent with number versus when they are all incongruent with number, a situation that can be thought of as an extreme difference in stimulus set protocol (DeWind et al., 2015). Thus, we hypothesized that applying the DeWind et al. (2015) model might reveal significant inter-test reliability when applied to the Clayton et al. (2015) data set. Furthermore, to the extent that different protocols do induce differences in the ANS, our model provides separate measures of numerical acuity and the biasing effect of non-numerical features allowing for a more quantitative assessment of those differences.

## Materials and Methods

### Data Set

All the analyses and findings are the result of reanalysis of two previously collected data sets: Clayton et al. (2015)^1^ and DeWind et al. (2015). The DeWind data set contained 20 participants who completed at least 750 trials. The Clayton data set consisted of 57 participants who completed two blocks of the Panamath protocol (60 trials each) and two blocks of the Gebuis and Reynvoet (G&R) protocol (96 trials each).

### Stimuli

**FIGURE 1:**
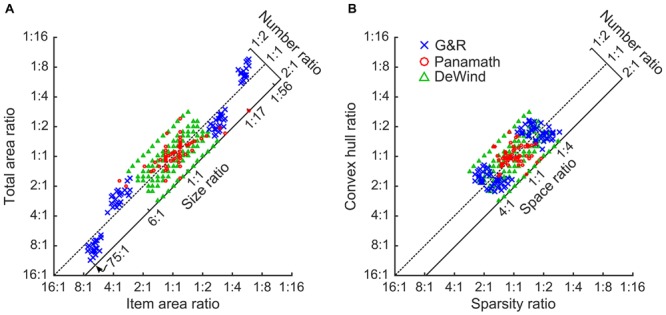
**Stimulus Space.** All stimulus pairs plotted according to the left to right ratio of item area against total area **(A)**, and sparsity against convex hull **(B)**. The diagonal dotted line shows where stimulus pairs of equal numerosity would appear. Pairs farther up and to the left of both plots have a smaller left to right numerical ratio, and those down and to the right have a larger left to right numerical ratio. Pairs farther down and left have a smaller size **(A)** or spacing **(B)** ratio, and those farther up and to the right have a larger size **(A)** or spacing **(B)** ratio. Red circles are Panamath pairs, blue “x”s are G&R pairs, and green triangles are DeWind pairs.

**Figure 1** illustrates the ratios for numerosity and non-numerical features used by the two stimulus sets tested in Clayton et al. (2015) and the stimulus set used in DeWind et al. (2015). The axes highlight the relevant aspects of the stimuli from the perspective of the modeling approach used here. On the x-axis are the “intrinsic” features (Dehaene et al., unpublished manuscript), those that pertain to the individual items in the array. These are the average item area, and “sparsity.” Sparsity is the amount of space in the array per item (convex hull area divided by number of items). On the *y*-axis are the “extrinsic” features, total area and convex hull. These are features of the array as a whole and are dependent on the number of items. By definition each extrinsic feature is equal to the corresponding intrinsic feature multiplied by the number of items. Thus, on a log scaled plot like **Figure 1** the numerical ratio of the arrays can be read out from its position on the plot. The further up and left a stimulus pair, the smaller left-to-right the numerical ratio. When plotted in this way we can also see the orthogonal axes, which we can think of as two additional stimulus features. We refer to the orthogonal features as “size,” for the feature related to total area and item area and “spacing” for the feature related to sparsity and convex hull (DeWind et al., 2015). Size represents the aspect of the stimulus that changes when a fixed number of dots change size, thus changing both total area and item area. Spacing represents the aspect of the stimulus that changes when a fixed number of dots are spread out or contracted together, thus changing both convex hull and sparsity.

As can be seen in **Figure 1** and in the summary statistics in **Table 1** the protocols differ in many respects. Panamath has the smallest range in all non-numerical feature ratios, but a larger range of numerical ratios compared to G&R. Partly as a result of this tradeoff G&R non-numerical feature ratios are less correlated with numerical ratio than Panamath. The DeWind stimuli fall between the Panamath and G&R stimulus sets in both these regards with the same maximum numerical ratio as Panamath (1:2), intermediate variance in non-numerical feature ratios, and intermediate correlation between numerical and non-numerical feature ratios. The notable features of the DeWind stimulus set are the orthogonalization of number, size, and spacing ratios and the orderly sampling of the stimulus space. These features optimize model fit and decrease the confidence intervals on the coefficient estimates, but are not necessary to fit the model. Indeed, there is nothing special about the stimuli in DeWind et al. (2015); all the insights of that paper depend on the analysis approach, which can be applied to other stimulus protocols (assuming that no non-numerical feature is perfectly correlated with number).

**Table 1:** Comparison of stimulus feature ratio statistics.

Protocol	Statistic	Number	Total Area	Item Area	Convex Hull	Sparsity	Size	Spacing
Panamath	Correlation with number (r)	1.00	-0.04	-0.68	0.52	-0.74	-0.43	-0.23
	Ratio range	1.06–2.00	1.00–2.93	1.00–5.86	1.00–1.78	1.00–1.89	1.00–17.2	1.01–2.23
	Mean ratio	1.3	1.3	1.6	1.2	1.3	2.1	1.4

G&R	Correlation with number (r)	1.00	0.38	0.18	0.20	-0.36	0.28	-0.09
	Ratio range	1.14–1.64	1.64–11.1	2.34–7.40	1.19–2.36	1.04–2.91	4.38–74.7	1.77–5.18
	Mean ratio	1.3	4.3	4.3	1.7	1.7	18.2	3.0

DeWind	Correlation with number (r)	1.00	0.44	-0.44	0.44	-0.44	0.00	-0.01
	Ratio range	1.12–2.00	1.00–2.83	1.00–2.83	1.00–2.83	1.00–2.83	1.00–4.00	1.00–4.00
	Mean ratio	1.4	1.5	1.5	1.5	1.5	2.1	2.1

*Correlations with number are taken from the log of the feature ratios (e.g., log of number ratio correlated with log of total area ratio for all pairs in a stimulus set). Mean ratios are calculated on a log scale (geometric mean).*

### Modeling Overview

The numerical ratio between two arrays is known to strongly affect accuracy for non-symbolic numerical comparison: a 1:2 ratio is easier than a 5:6 ratio. The Weber fraction (*w*) is often used to account for the ratio effect, summarizing performance across the range of numerical ratios (Piazza et al., 2010). Non-numerical features such as the total surface area of the items or the area of the convex hull might also affect accuracy. Non-numerical features also differ by a ratio. For example, total area may differ between arrays by a 1:2 ratio while number differs by a 2:3 ratio. Our modeling approach, developed in DeWind et al. (2015), is designed to simultaneously assess the roles of numerical ratio and non-numerical ratios on accuracy. It can be thought of as an extension of the Piazza et al. (2010) model. Where Piazza et al. (2010) use numerical ratio as the only regressor in a generalized linear model of accuracy, we include more regressors to account for the non-numerical feature ratios.

The situation is complicated by the complete collinearity of some combinations of stimulus features. For any combination of an intrinsic feature, extrinsic feature, and number (three variables) there are only two degrees of freedom. For example, if we set the total area of a stimulus and the average item area of a stimulus, then we cannot freely set the number of items, because it is already mathematically determined (number equals total area divided by average item area). If we tried to fit a regression using the ratio of these three features as regressors, there would be no unique solution to the linear equations. Variance in accuracy could be attributed to number or to a combination of total area and item area. This is where the “orthogonal” stimulus features, size and spacing, are useful. They represent the single degree of freedom left to each pair of intrinsic and extrinsic features after number has been determined.

Consequently we constructed a simple generalized linear model of approximate number comparison with three predictors: number ratio, size ratio and spacing ratio. The number ratio coefficient is a measure of acuity and its reciprocal is proportional to *w*. The size and spacing coefficients summarize the effect of the non-numerical features. A positive size coefficient means participants perceive larger dots as more numerous, all other things being equal. A negative size coefficient means smaller dots are perceived as more numerous. A positive spacing coefficient means more spaced out dots are perceived as more numerous, negative that more densely packed dots are perceived as more numerous.

### Modeling (Equations and Methodological Details)

We fit the model to different subsets of the Clayton et al. (2015) data set depending on the analysis as described in the results. In one analysis we collapsed across participant, but ran the model separately for each protocol, in another we separated by participant and protocol, and in a third we separated by participant, protocol, and block. Here we provide the formula for the model and the equations for the size and spacing parameters. Further details can be found in DeWind et al. (2015).

(1) $$\log_{2}(\mathrm{Size})=\log_{2}(\mathrm{TSA})+\log_{2}(\mathrm{ISA})$$

(2) $$\log_{2}(\mathrm{Space})=\log_{2}(\mathrm{CH})+\log_{2}(\mathrm{Spar})$$

Where TSA is the total surface area of all items in an array, ISA is the area of an individual item or the mean area if the items are heterogeneous, CH is the convex hull of an array, and Spar is the sparsity defined as the convex hull divided by the number of items.

We fit a generalized linear model of choice probability with three predictor variables (log_2_ of the ratios of number, size, and space), a probit link function, and a binomial error distribution. The equation is:

(3) $$p(\mathrm{ChooseRight})=\frac{1}{2}\;\left[1+\mathrm{erf}\left(\frac{\;\beta_{\mathrm{side}}+\beta_{\mathrm{num}}\log_{2}(r_{\mathrm{num}})\;+\;\beta_{\mathrm{size}}\log_{2}(r_{\mathrm{size}})\;+\;\beta_{\mathrm{space}}\log_{2}(r_{\mathrm{space}})}{\sqrt{2}}\right)\right]$$

or simplified as:

(4) $$p(\mathrm{ChooseRight})=\Phi (\beta_{\mathrm{side}}+\beta_{\mathrm{num}}\log_{2}(r_{\mathrm{num}})+\beta_{\mathrm{size}}\log_{2}(r_{\mathrm{size}})+\beta_{\mathrm{space}}\log_{2}(r_{\mathrm{space}}))$$

where Φ is the cumulative normal distribution; r_num_, r_size_, and *r*_space_, refer to the ratio of number, size, and spacing of the array presented on the right to the number, size, and spacing of the array presented on the left; p(ChooseRight) is the probability of choosing the stimulus presented on the right; and erf is the error function. The intercept, β_side_, captures the tendency to choose the stimulus presented on the right regardless of its features. The model was fit using the Matlab statistics and machine learning toolbox.

The Weber fraction (*w*) can be calculated from β_num_ in a way that is analogous to other logarithmic models of numerical comparison (Piazza et al., 2010).

(5) $$w=\frac{1}{\sqrt{2}\;\beta_{\mathrm{num}}}$$

A lapse parameter that appeared in the model developed in DeWind et al. (2015) was excluded from the model here. The lapse parameter was originally used to estimate the percentage of trials on which a participant’s response was a guess and unrelated to any stimulus features. Previous unpublished work in our lab demonstrated that to get good coefficient estimates and also estimate the lapse rate very easy numerical ratios must be tested or a large number of trials must be administered, preferably both. Since the dataset collected by Clayton et al. (2015) did neither, we were unable to include the lapse parameter for this analysis.

We also note a difference in terminology between this report and DeWind et al. (2015). In that paper we used the term field area and here we model “convex hull.” Convex hull is the area of the smallest convex polygon that contains all the items in an array. Field area is a very closely related parameter: it is the area of an invisible circle within which the array generation algorithm used by DeWind et al. (2015) placed the items. Although we could not extract the convex hull from the DeWind data set, we constructed 10,000 new stimuli using the same method and found a correlation of 0.93 between convex hull and field area. Because these features reflect very similar aspects of the stimulus, we have treated field area as convex hull for the purposes of comparing our stimuli with the Panamath and G&R protocols.

### Exclusion Criteria

Clayton et al. (2015) excluded ten participants that did not perform significantly above chance on one or more of the four blocks. However, DeWind et al. (2015) showed that some participants perform at or below chance when the numerical ratio is particularly difficult and they are biased by non-numerical features of the stimuli. In other words, if a non-numerical feature such as convex hull differs between two stimuli by a large ratio and the numerical ratio is very small, then an influence of convex hull on behavior could induce participants to consistently choose the wrong stimulus (see “incongruent” condition in Figure 3B in DeWind et al., 2015). Indeed, the G&R stimulus set is notable for relatively large ratios of non-numerical features compared to number. As a result, below or at chance performance is not necessarily an indication that a participant is guessing but instead could reflect strong bias from another stimulus feature, an effect that can be captured by our model. Our reanalysis therefore excluded only three participants for whom the whole model failed to explain significant variance in choice behavior for one or more blocks (log likelihood test, *p* > 0.05), or who exhibited no evidence that they used number in one or both protocols (*t*-test for β_num_, *p* > 0.05).

## Results

### The DeWind et al. (2015) Model Fits the Clayton et al. (2015) Data

**FIGURE 2:**
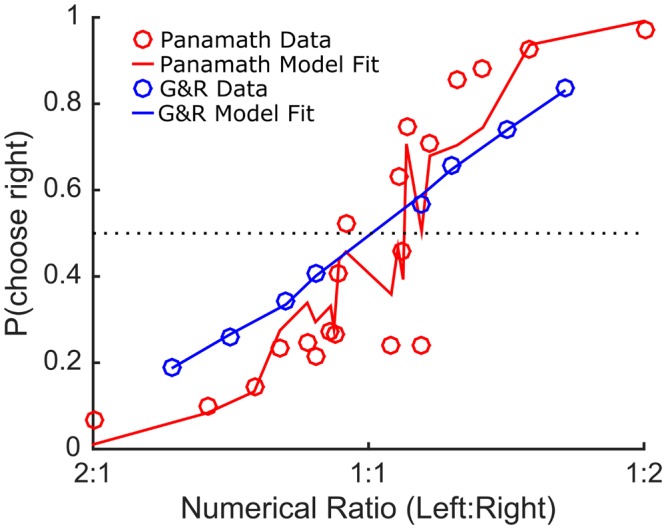
**DeWind et al. (2015) model fits the data.** Proportion of trials on which participants choose the array on the right side plotted against the left to right numerical ratio (irrespective of non-numerical feature ratio) for Panamath (red circles) and G&R (blue circles). The line shows the predictions of the model fit separately to the data for each protocol collapsing across participant.

**Figure 2** shows how the DeWind et al. (2015) model fit the data for the protocols in the Clayton et al. (2015) data set (collapsed across participant). Both fits were highly statistically significant (log likelihood ratio test: Panamath, *p* ≪ 0.001; G&R, *p* ≪ 0.001). However, it is clear that the model fits data from the G&R protocol better than the Panamath protocol. Indeed, the Panamath protocol data and fit-line follow a tortuous path across numerical ratios, because Panamath does not systematically control for convex hull, which has an important effect on performance. As a result, some Panamath numerical ratios are more congruent with convex hull ratio on average, while others are more incongruent. The model, accounting for the effect of convex hull, predicts some of these deviations, however, it does not fully account for them, given that the fit line does not cross each data point as it does with the G&R stimuli. This may reflect noise given that Panamath was tested with fewer trials and more numerical ratios than G&R and therefore fewer samples make up each point.

### Inter-test Reliability

**FIGURE 3:**
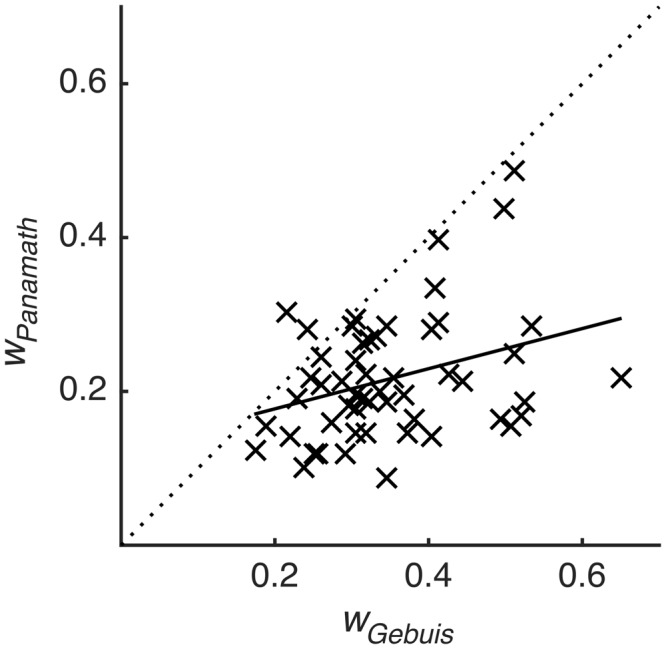
**Weber fraction is correlated across protocol.** *w* calculated for each participant from the Panamath blocks plotted against *w* calculated from the G&R blocks. Dotted line is unity, and solid line is the best-fit linear regression (*r* = 0.335, *p* = 0.013).

We next examined the main finding of Clayton et al. (2015) using our model. Clayton et al. (2015) found that accuracy was not significantly correlated within subjects across protocols (although it was trending in that direction). We fit each participant’s data for the two protocols using the model described above (collapsing across block), extracted the coefficients from the fit, and calculated *w* from Eq. 5. This *w* coefficient is a better measure of numerical acuity than *w* derived from other methods and accuracy itself, because it is relatively insulated from the influence of non-numerical stimulus features (DeWind et al., 2015). We found that *w*, so calculated, was significantly correlated within subjects across protocols (*r* = 0.335, *p* = 0.013; **Figure 3**). **Table 2** shows the inter-test reliability of all the coefficients produced by the model. Note that β_num_ and *w* have very similar inter-test reliability; they are virtually the same metric, one being proportional to the reciprocal of the other. However, it is also clear that Panamath *w* were lower than G&R *w*, a difference we will examine in more detail below.

**Table 2:** Inter-test reliability.

	β_num_	β_size_	β_space_	*w*
*r (p)*	0.333 (0.014)	0.054 (0.697)	0.484 (<0.001)	0.335 (0.013)

*Correlation coefficients between values calculated from Panamath blocks and from G&R blocks.*

### Test–retest Reliability

We also assessed the within protocol test–retest reliability of the model coefficients. For each of the two stimulus protocols we refit the model to each block for each participant and measured the correlation across blocks (**Table 3**). Using Weber fraction derived from our model (or β_num_) we failed to observe the superior test–retest reliability for the G&R protocol that Clayton et al. (2015) observed. We did, however, find that Panamath reliability is very low for β_size_ and did not meet statistical significance. This is likely the reason that inter-test reliability was so low for β_size_; if a metric is uncorrelated with itself it is unlikely to be correlated with anything else. Why Panamath yields such unreliable estimates of the influence of dot size on numerosity judgments is not immediately obvious.

**Table 3:** Test–retest reliability.

	β_num_	β_size_	β_space_	*w*
Panamath (60 trials)	0.443 (<0.001)	0.246 (0.073)	0.451 (<0.001)	0.468 (<0.001)
G&R (96 trials)	0.465 (<0.001)	0.722 (<0.001)	0.673 (<0.001)	0.350 (<0.009)
DeWind (375 trials)	0.731 (<0.001)	0.771 (<0.001)	0.654 (0.002)	0.675 (0.001)

*Correlation coefficients between values calculated from block one and block two separately for each protocol. *p*-values in parentheses.*

We next performed the same test–retest reliability analyses on the dataset collected by DeWind et al. (2015). We divided the 750 trials that participants completed into two consecutive artificial pseudo-blocks of 375 trials each. We fit the model to each block and measured the correlation of the resulting coefficients. β_num_ reliability from the DeWind data set was higher than Panamath and G&R, but did not differ significantly (Fisher r-to-z transform: Panamath one-tailed *p* = 0.053; G&R one-tailed *p* = 0.064).

It is important to note that the length of a test plays a critical role in its reliability. The reliability estimates given in **Tables 2** and **3** reflect the reliability on only one block for each protocol. The Spearman–Brown formula allows us to estimate reliability of the whole test, if by whole test we mean administering two blocks of each protocol or, in the case of inter-test reliability, administering all four blocks of the G&R and the Panamath protocol. **Table 4** presents these corrected reliability correlations.

**Table 4:** Corrected reliability.

	β_num_	β_size_	β_space_	*w*
G&R + Panamath	0.533	0.094	0.684	0.582
Panamath (120 trials)	0.614	0.395	0.622	0.638
G&R (192 trials)	0.635	0.839	0.805	0.519
DeWind (750 trials)	0.845	0.871	0.791	0.806

*The first row shows the same values from **Table 2** corrected for attenuation. These are estimated inter-test reliabilities assuming no measurement error. The other three rows show the same values from **Table 3** corrected for the number of trials using the Spearman–Brown formula. These values represent the estimated reliability for actual number of trials administered (as opposed to the reliability of each split-half). *p*-values are as in **Tables 2** and **3**.*

### Acuity and Bias Differ Systematically between Protocol

**FIGURE 4:**
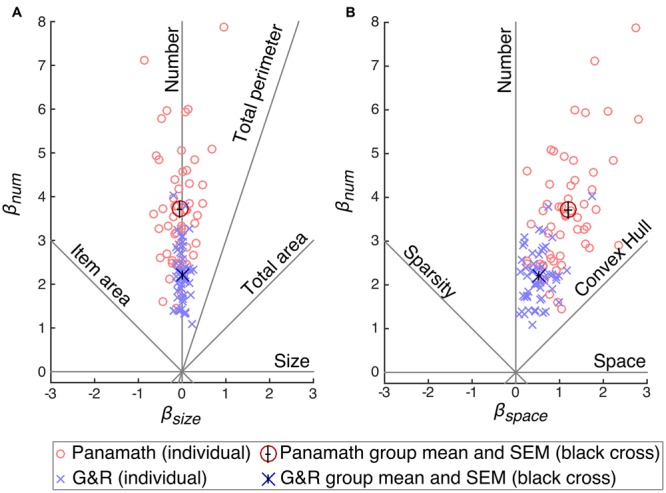
**Coefficient space reveals differences in acuity and bias between protocol.** Model coefficients were calculated for each participant separately for each condition and plotted against each other: β_num_ × β_size_ **(A)** and β_num_ × β_space_ **(B)**. Red circles show coefficients calculated from Panamath trials and blue “×”s from G&R. The larger and darker red circle and blue “×” show the means for each protocol, and the small black cross in the center of these symbols indicates the SEM. Acuity is equivalent to the height of a point and bias is indicated by its deviation from the vertical “Number” feature line.

**Figure 4** shows the coefficients calculated for each participant in each protocol (collapsed across block) plotted against each other. **Figure 4A** shows β_num_ plotted against β_size_, and **Figure 4B** shows β_num_ against β_space_. A hypothetical unbiased participant would have a positive β_num_ and zero β_size_ and zero β_space_. Such a participant would fall on the “Number” feature line in **Figures 4A, B**. The height of the point (the magnitude of β_num_) is an indication of acuity in discriminating number; larger β_num_ results in smaller *w*. Participants biased by non-numerical visual features will deviate from the number feature line in one or both of the plots. The degree of the deviation indicates non-numerical feature bias.

The other feature lines in **Figures 4A, B** (e.g., convex hull, item area, etc.) show the position of a hypothetical participant that discriminated stimuli on the basis of that feature rather than number. For example, if a participant always and only chose the stimulus that had a larger convex hull, we would expect her to fall along the convex hull feature line in **Figure 4B** (β_num_ and β_space_ would be equal and positive and β_size_ would be zero). The further from the origin the point on the convex hull feature line, the greater her acuity in discriminating convex hull.

**Table 5** shows the means and standard deviations of the parameter estimates of the model fit to each protocol for each participant (collapsing across block). The group mean of β_num_ (Panamath *t* = 20.4, *p* ≪ 0.001; G&R *t* = 25.4, *p* ≪ 0.001) and β_space_ (Panamath *t* = 15.1, *p* ≪ 0.001; G&R *t* = 12.5, *p* ≪ 0.001) are significantly different from zero, however, β_size_ was not (Panamath *t* = -1.3, *p* = 0.20; G&R *t* = 0.4, *p* = 0.67). This means that overall, participants perceived more spaced out dots to be more numerous, but were indifferent to the size of the dots. Mean coefficient points were closer to the number feature line than to the convex hull feature line indicating that number was the primary determinate of participants’ choices in both protocol conditions (β_num_ significantly greater than β_space_ : Panamath paired *t* = 16.4, *p* ≪ 0.001; G&R paired *t* = 20.4, *p* ≪ 0.001). Thus, although participants were biased by spacing, they still utilized number more than any non-numerical feature.

**Table 5:** Mean and standard deviation of the parameter estimates.

	β_num_	β_size_	β_space_
Panamath	*M* = 3.71, *SD* = 1.34	*M* = -0.06, *SD* = 0.33	*M* = 1.20, *SD* = 0.58
G&R	*M* = 2.21, *SD* = 0.64	*M* = 0.01, *SD* = 0.10	*M* = 0.52, *SD* = 0.31

*The model was fit to each participant’s data separately for each protocol, but collapsing across block. These are the same means plotted in **Figure 4**.*

Although participants showed the same overall pattern of bias in both protocols, there were significant differences. The coefficient weights for number and spacing were significantly greater in Panamath than in G&R (paired *t* = 8.65, *p* ≪ 0.001; paired *t* = 9.69, *p* ≪ 0.001 respectively). This explains the lower *w* observed for the Panamath protocol in **Figure 3**; overall, participants had higher acuity in the Panamath condition. However, the larger coefficient for spacing in Panamath indicates that they were also more biased toward spread out dots.

It is not immediately obvious which weighting of stimulus features is better. As shown by the average β_num_ and β_space_ values in **Figure 4**, Panamath stimuli result in higher acuity but more spacing bias and G&R stimuli yield lower acuity with lower bias. One possibility is that participants tuned their response strategy to reflect the fact that convex hull is more correlated with number in the Panamath stimulus set compared to the G&R stimulus set. To test this hypothesis we generated predicted responses on the G&R trials with the mean coefficients fit to the Panamath protocol and vice-versa. In other words, we simulated what would happen if participants had responded with the acuity and bias of the opposite protocol condition. We found that participants were better off with the acuity and bias induced by the G&R protocol regardless of which stimulus set they were discriminating (**Table 6**). Thus the trade-off of higher acuity but more bias induced by the Panamath protocol does not appear to be beneficial given that despite lower *w*, over-reliance on convex hull results in poorer performance.

**Table 6:** Alternative strategy analysis.

	Actual accuracy	Predicted accuracy with alternative acuity and bias (according to model)
Panamath stimulus set	71.7%	78.3% (with G&R acuity and bias)
G&R stimulus set	70.1%	62.5% (with Panamath acuity and bias)

*Estimated accuracy on each protocol if participants had had the same acuity and bias calculated from the other protocol.*

Finally, we investigated the time course of the protocol induced differences in acuity and bias by analyzing the coefficients separately fit to each block and each participant. We were interested in both primacy effects depending on which protocol was viewed first and learning effects across presentations of the same protocol. We ran three repeated measures ANOVAs predicting each of the three model coefficients with fixed effects for time (first or second presentation of a protocol), protocol presentation order (Panamath first or G&R first), and protocol itself. Consistent with our previous analysis we found an effect of protocol on β_num_ [*F*(1,215) = 100.53, *p* ≪ 0.001] and β_space_ [*F*(1,215) = 111.39, *p* ≪ 0.001], but not for β_size_ [*F*(1,215) = 2.79, *p* = 0.097]. There was also a significant effect of presentation order on β_num_[*F*(1,215) = 6.11, *p* = 0.017], but not β_size_ or β_space_ (both *p* > 0.1). Examination of the data showed that β_num_ was suppressed when participants were exposed to G&R first compared to Panamath first, and that this deficit was sustained across protocols throughout the experiment. There was no effect of time, indicating that there was no significant practice effect between the first and second administration of either protocol (all *p* > 0.1). Thus, both protocol and order of protocol affected stimulus feature weighting with the first protocol leaving an impression on performance that remained for at least the duration of the experiment.

## Discussion

We reanalyzed data collected by Clayton et al. (2015) and found that ANS acuity as measured by *w* derived from the DeWind et al. (2015) model showed significant inter-test reliability across stimulus protocols. Clayton et al. (2015) and Smets et al. (2015) found no significant correlation between accuracy in different stimulus protocols. Clayton et al. concluded that, “…dot comparison tasks created with protocols used by different research groups do not appear to be measuring the same construct.” Smets et al. (2015) similarly interpret the lack of correlation in performance as evidence against the idea that ANS can be reliably measured. Using the DeWind et al. (2015) model, we find instead that *w* is indeed significantly correlated across the Panamath and G&R protocols. We conclude that overlapping cognitive mechanisms underlie performance in both protocols.

Although the correlation in *w* was significant across protocol the correlation is not very strong, a point Clayton et al. (2015) rightly emphasize with regards to accuracy. It is important to note that test–retest reliability can impose an upper limit on inter-test reliability and thus test validity; two tests cannot measure the same thing as each other if they do not consistently measure anything at all. Clayton et al. (2015) point out that accuracy obtained from Panamath has low test–retest reliability, even after accounting for the relative number of trials. Using our modeling approach we found that β_num_ and *w* reliability were relatively similar and low across G&R and Panamath. β_size_ from Panamath, however, was not significantly reliable whereas it was from G&R; this likely contributed to the low inter-test reliability for β_size_. The DeWind protocol had better reliability for β_num_ and *w*, although the trend was not significant. In general, the effect of protocol on reliability remains tenuous; if some protocols are more reliable it is likely due to testing a larger range of numerical and non-numerical ratios and decorrelating them from each other to the extent possible. However, it is likely that the total number of trials in the assessment has an equal or perhaps larger effect on test-retest reliability and thus perhaps also on inter-test reliability. The length of a test is known to influence its reliability, and reliability can be improved with repeated administration of the same items. Indeed, the number of trials in an ANS protocol similar to Panamath was recently found to have a profound and significant effect on reliability, with hundreds of trials required for acceptable reliability (Lindskog et al., 2013). The same study also found that a task that adapts ratio difficulty to individual participants’ skill level gets good reliability in fewer trials. An important future direction will be to empirically determine if protocols with greater test-retest reliability, especially those using more trials, show greater inter-test reliability than what we find here and what Clayton et al. (2015) and Smets et al. (2015) found.

Without doubt studies examining the correlation between performance on an ANS task and mathematical achievement will lose power if the ANS assessment is too short to produce a reliable *w* and improving reliability in assessments of ANS acuity is an important goal for this research program. In some cases, however, it may not be feasible to run many trials, and reliability will necessarily be low. In these cases, low reliability can be ameliorated by a larger sample of individuals. In this case the ability to predict math performance of a given individual may be very low, but correlations may still be apparent at the group level (e.g., Halberda et al., 2012).

Although *w* and the other model coefficients that estimate bias were correlated across the two protocols, we also found statistically significant protocol induced differences. In particular, the Panamath protocol induced a larger reliance on the spacing of dots but also better numerical acuity, whereas the G&R protocol resulted in greater focus on number itself, albeit at lower acuity. Thus, our reanalysis of Clayton et al. (2015) supports their original conclusion that different stimulus protocols affect ANS measurement. An important question for future research is whether protocol induced changes in bias and acuity affect the correlation between ANS and mathematics.

There are two possible explanations for the differences in coefficients that we observe here. They could be “item” effects, caused by differences in the stimulus pairs on the current trial, or they could be “context” effects caused by differences in the pairs seen up to that point. The difference is subtle but is made clearer by a hypothetical: if probe trials were included in both protocols that were visually identical, would the responses be identical regardless of the protocol in which they were embedded? If so then the effects we report here are purely item effects, if not then the effects are at least partially contextual.

We know that item effects on accuracy exist; numerical, size, and space ratios all affect performance. However, our analysis approach attempts to account for as much of the item effect as possible, and our previous work demonstrated that item effects, as exemplified by non-numerical features being either fully congruent or fully incongruent with number, on coefficient estimates were negligible (DeWind et al., 2015). Thus, we think it is likely that the differences in coefficients that we report here are primarily a function of context rather than item. Furthermore, our between-subject finding that protocol administration order affected performance on later protocols demonstrates that at least some effects were contextual: performance on the exact same trials in the same protocol depended on which stimuli participants previously had been exposed to.

The time course of the differences reported here is worth considering. We observed no difference in performance between the first and second administration of each protocol, thus we can conclude that the contextual effects must establish themselves within a few dozen trials. This result is reminiscent of the findings by Odic et al. (2014) of experimental hysteresis whereby measures of *w* are lower when participants are given easier trials at the outset of a numerical comparison task (Odic et al., 2014). In addition to the context effect established by the first protocol, which lasted throughout the experiment, we also found strong effects of the current protocol, suggesting that participants continuously adapt their responses to the current stimuli. There is evidence from the animal and human learning literature that such changes may occur in steps rather than gradually (Gallistel et al., 2004; Gaschler et al., 2015), and more sophisticated analysis approaches might be able to tease out the temporal onset of these effects.

Finally, future work will also need to assess exactly which stimulus features affect bias and acuity. The G&R and Panamath protocols differ along every dimension we considered in **Table 1**, and, of course, these differences were not controlled to allow for a careful analysis of their effects. However, we can make some educated hypotheses regarding the features that play the most important role. The biggest differences between the protocols both in terms of the range of ratios and in terms of the correlation with number were those related to the size of the items. However, β_size_ did not change between protocols, and across subjects it was close to zero. β_space_ exhibited differences across protocol, and also was significantly positive overall. Thus, it seems likely that small differences in the correlation between convex hull and number play an important role in shaping the behavioral profile of participants in numerical discrimination tasks in general.

## Conclusion

Contrary to the findings of Clayton et al. (2015), we found that the Panamath protocol and the G&R protocol show significant inter-test reliability. Nevertheless, inter-test reliability was low, likely due to low test–retest reliability. We recommend using more trials to increase test–retest reliability which should ultimately increase inter-test reliability. In agreement with Clayton et al. (2015) we find that the two protocols influenced the acuity and bias of participants. Panamath induces better acuity but also a greater reliance on convex hull, whereas G&R focuses attention to number, but at the cost of lower acuity. Thus more research is needed to understand how contextual factors such as the range of stimulus parameters influence discrimination and how this affects our study of the relationship between the ANS and symbolic mathematical abilities.

## Author Contributions

ND analyzed the data, and ND and EB wrote the manuscript.

## Conflict of Interest Statement

The authors declare that the research was conducted in the absence of any commercial or financial relationships that could be construed as a potential conflict of interest.

## Footnotes

1 The Clayton et al. (2015) data set is available for download at http://dx.doi.org/10.6084/m9.figshare.1546747
